# Elucidating the role of hepatic enzymes in spontaneous abortion: a Mendelian randomization approach

**DOI:** 10.3389/fgene.2024.1336728

**Published:** 2024-09-02

**Authors:** Yingping Zhu, Zhenghong Li, Xingfang Liu, Chengping Wen

**Affiliations:** ^1^ The First Affiliated Hospital of Zhejiang Chinese Medical University (Zhejiang Provincial Hospital of Chinese Medicine), Hangzhou, China; ^2^ Research Department, Swiss University of Traditional Chinese Medicine, Bad Zurzach, Switzerland; ^3^ College of Basic Medical Science, Zhejiang Chinese Medical University, Hangzhou, China; ^4^ Key Laboratory of Chinese Medicine Rheumatology of Zhejiang Province, Hangzhou, China

**Keywords:** spontaneous abortion (SA), aspartate aminotransferase (AST), alanine aminotransferase (ALT), Mendelian randomization (MR), hepatic enzymes

## Abstract

**Background:**

While the hepatic enzymes Aspartate Aminotransferase (AST) and Alanine Aminotransferase (ALT) are crucial for liver function, their role in Spontaneous Abortion (SA) has not been thoroughly explored. Utilizing Mendelian Randomization (MR), this study aims to clarify the putative causal relationship between AST/ALT levels and SA.

**Methods:**

Genome-wide association study (GWAS) summary data for SA (finn-b-O15_ABORT_SPONTAN), AST (ukb-d-30650_raw), and ALT (ukb-d-30620_raw) were acquired from the Integrative Epidemiology Unit OpenGWAS database. Bidirectional MR analysis was conducted using MR-Egger, Weighted Median, Simple Mode, Weighted Mode, and Inverse Variance Weighted (IVW) algorithms, and the robustness of MR results was assessed through sensitivity analyses including Heterogeneity, Horizontal Pleiotropy, and Leave-One-Out (LOO) tests. The causal role of AST and ALT’s coaction in SA was explored via multivariable MR (MVMR) analysis.

**Results:**

The MR results via the IVW algorithm revealed a causal relation between both AST and ALT and SA (AST: *P* = 0.013; ALT: *P* = 0.017), identifying them as risk factors for SA (AST: odd ratio (OR) = 1.019; ALT: OR = 1.012). Sensitivity analysis substantiated the reliability of these results. Moreover, not notably causality was found between SA and AST/ALT (*P* > 0.05). Through MVMR analysis, AST and ALT demonstrated functional complementarity in the occurrence of SA, attributable to counterbalanced causalities (AST: *P* = 0.128; ALT: *P* = 0.899).

**Conclusion:**

The study substantiates a causal linkage between transaminase levels and SA, enhancing our understanding of their biological interaction and the regulatory mechanisms at play. These insights could have implications for identifying novel biomarkers and therapeutic targets for SA.

## 1 Introduction

Pregnancy loss, also known as miscarriage or spontaneous abortion (SA), is an unintended pregnancy loss that occurs before the embryo reaches viability, usually within the first 20 weeks of pregnancy (ACOG, 2018), and our definition of SA refers to termination of pregnancy within 28 weeks of gestational age, with a fetal mass of no more than 1 kg (NICE, 2023). Clinical manifestations are bleeding and spasmodic pain. SA can be divided into uncomplicated and accompanied complications, with common complications being massive bleeding and infection. Uncomplicated bleeding and cramping pain are the most common symptoms in patients with pregnancy loss. Other symptoms of uncomplicated pregnancy loss may include loss or reduction of pregnancy reactions, such as decreased breast tenderness and/or decreased nausea and vomiting (DeVilbiss et al., 2020). The occurrence of SA can be influenced by a number of factors, including genetic factors, embryo abnormalities, maternal health problems, chronic diseases, substance abuse, exposure to harmful substances, and intrauterine infections (Nguyen et al., 2009). Miscarriage is usually accompanied by vaginal bleeding and abdominal pain, but there may be no noticeable symptoms (Deng et al., 2022; Alves and Rapp, 2023). About 10% of women of childbearing age experience SA (Kostova et al., 2023), and about 1%–3% experience recurrent miscarriage (van Wely, 2023), which has a huge impact on women’s physical and mental health, as well as a serious economic burden on the family and society (Quenby et al., 2021), so it is important to find the risk factors for RA prevention and intervene early.

Some studies have indicated a potential association between altered levels of aspartate aminotransferase (AST) and alanine aminotransferase (ALT) and the occurrence of SA. A study of women patients at risk for miscarriage and at risk for preterm labor found that AST levels were significantly higher in patients at risk for miscarriage than in a group of normal pregnant women, but there was no significant difference in ALT levels (Micle et al., 2012). ALT is produced primarily in the plasma of hepatocytes, and is a specific marker of liver injury, whereas AST is found in the liver, heart, and other tissues (Cooper et al., 2023), and elevated levels of both in the blood are indicative of liver injury or disease (Tamber et al., 2023). High levels of AST and ALT may reflect an underlying inflammatory state, and inflammation has been recognized to be strongly associated with the development of SA (Gao et al., 2020). Severe liver injury may affect maternal metabolism and hormonal balance, which may affect embryonic development (Obiegbusi et al., 2023). AST and ALT are involved in oxidative stress, which has been implicated in the development of SA (García-Romero et al., 2019). Raising a noteworthy question: Do the level changes of AST and ALT directly influence the risk of SA? We hypothesize that an abnormal increase in AST and ALT may be related to some pathophysiological processes associated with SA, such as inflammation, oxidative stress, or vascular dysfunction. Furthermore, considering that AST and ALT are typically biomarkers of liver function impairment, it is also worth exploring whether they indirectly affect the risk of SA by influencing maternal metabolism or immune function.

Mendelian randomization (MR) is an analytical method used to study the causal relationship between observed biomarkers or environmental factors and a specific outcome, such as morbidity risk (Birney, 2022). In the study of disease mechanisms, with the discovery of a large number of genetic variants closely associated with specific traits in biology and the public release of data from many large-sample Genome-Wide Association Study (GWAS) (Wang et al., 2022), it has helped to reveal the intrinsic mechanisms of diseases (Bowden and Holmes, 2019).

To investigate the potential causal relationship between AST, ALT, and the risk of SA, this study conducted univariate and multivariate MR analyses on two independent samples.

## 2 Materials and methods

### 2.1 Data sources and summary

The summary data from GWAS for SA, AST, and ALT were obtained from the Integrative Epidemiology Unit (IEU) OpenGWAS database (https://gwas.mrcieu.ac.uk/). The data of SA (finn-b-O15_ABORT_SPONTAN) was comprised of 9,113 cases and 89,340 controls, with a total of 16,379,138 single nucleotide polymorphisms (SNPs). The numbers of SNPs for AST (ukb-d-30650_raw) and ALT (ukb-d-30620_raw) was 13,586,009 and 13,586,000, respectively. The MR method is based on three fundamental assumptions (ACOG, 2018): The genetic variants have a significant and direct association with the exposure being studied (NICE, 2023); The genetic variants are not influenced by any factors that could confound the relationship between the exposure and the outcome; and (DeVilbiss et al., 2020) The genetic variants solely affect the outcome through their impact on the exposure, without any additional effects on other outcomes. To simplify, these assumptions state that the genetic variants are strongly related to the exposure, unaffected by confounding factors, and have no other effects on the outcome.

### 2.2 GWAS data pre-processing

The “extract_instruments” function in R package “TwoSampleMR” was adopted to perform the data reading for exposure factors and filtering of instrumental variables (IVs), then the default p-value threshold of less than 5 × 10^−8^ in the MR analyses was applied to identify SNPs significantly associated with the exposure (Hemani et al., 2018) and to remove IVs with linkage disequilibrium (clump = TRUE, r2 = 0.001, kb = 10,000). Simultaneously, the IVs markedly relevant to the outcome were also rejected, and then the “harmonise_data” function was utilized to harmonize the effect equipotential with effect size. The exposure factors—IVs—outcome was matched for the following analysis. Finally, IVs were retrieved through PhenoScanner database, SNPs that might affect the outcome through other confounding factors were excluded to ascertain the ultimate SNPs as IVs. The IVs was evaluated by calculating the F-statistic, which was typically recommended to be 10 or higher to ensure the reliability of the genetic instruments used in MR analysis.

### 2.3 Bidirectional Mendelian randomization (MR) analysis and sensitivity analysis

Five algorithms uniting with the “mr” function of the R package “TwoSampleMR” were employed to execute the bidirectional MR analysis, including MR Egger (Jha et al., 2023), Weighted median (Soares et al., 2018), fixed effects Inverse variance weighted (IVW) (Zhang et al., 2023), Simple mode and Weighted mode (Llovet et al., 2019). Primarily, the MR results were referred to IVW and evaluated utilizing the Cochran Q value, derived from subsequent heterogeneity analysis. The scatter plots, forest plots and funnel plots were created to exhibit the results of causality between two aminotransferases and SA. Ultimately, the sensitivity analysis comprised of the Heterogeneity, Pleiotropy and Leave-One-Out (LOO) sensitivity test was executed to evaluate the reliability of the above MR results. First, Cochran’s Q test was used to evaluate the heterogeneity among SNPs, with p values greater than 0.05 indicating no heterogeneity. Secondly, horizontal pleiotropy was used to assess whether confounding factors existed between exposure factors and outcome sample data. The mr_pleiotropy_test function was used to test for the presence of horizontal polytropy in the evaluation, and the absence of horizontal polytropy indicated that the results were reliable. When the p-value is greater than 0.05, it indicates that there is no horizontal polytropy. Third, we conducted a LOO sensitivity test to determine whether there was an abnormal SNP that was sensitive to the outcome effect.

### 2.4 Multivariable MR (MVMR) analysis

**FIGURE 1:**
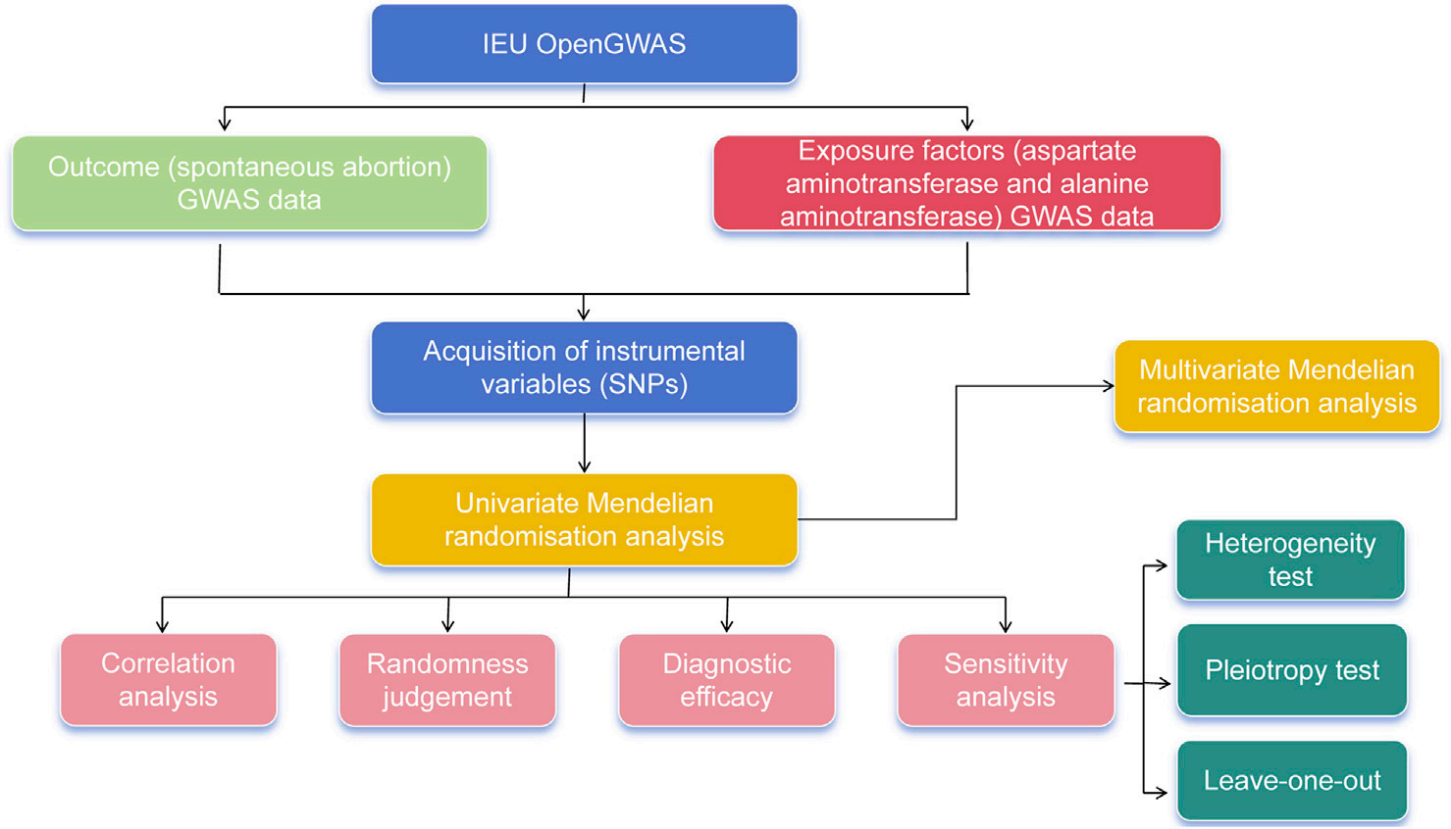
Flow chart of this study.

The “mv_extract_exposures” function in R package “TwoSampleMR” was adopted to perform the data reading for multivariable exposure factors and filtering of IVs (*P* < 5 × 10^−8^), followed by the elimination of IVs with linkage disequilibrium (clump = TRUE, r2 = 0.001, kb = 10,000). The “extract_outcome_data” function was also employed with proxies = TRUE and rsq = 0.8, and then the “mv_harmonise_data” function was utilized to harmonize the effect equipotential with effect size for MVMR analysis. The F statistic was calculated using the strength_mvmr function, with an F statistic greater than 10 indicating that genetic variation had sufficient power to correlate the exposure factor with the outcome. For analysis, this study mainly used the IVW method to explore the influence of two exposure factors on the outcome. In addition, pleiotropy_mvmr function was used to calculate horizontal pleiotropy in MVMR. The flow chart of this study is shown in Figure 1.

## 3 Results

### 3.1 Positive causal relationships of AST and ALT on SA occurrence based on bidirectional MR analysis

**FIGURE 2:**
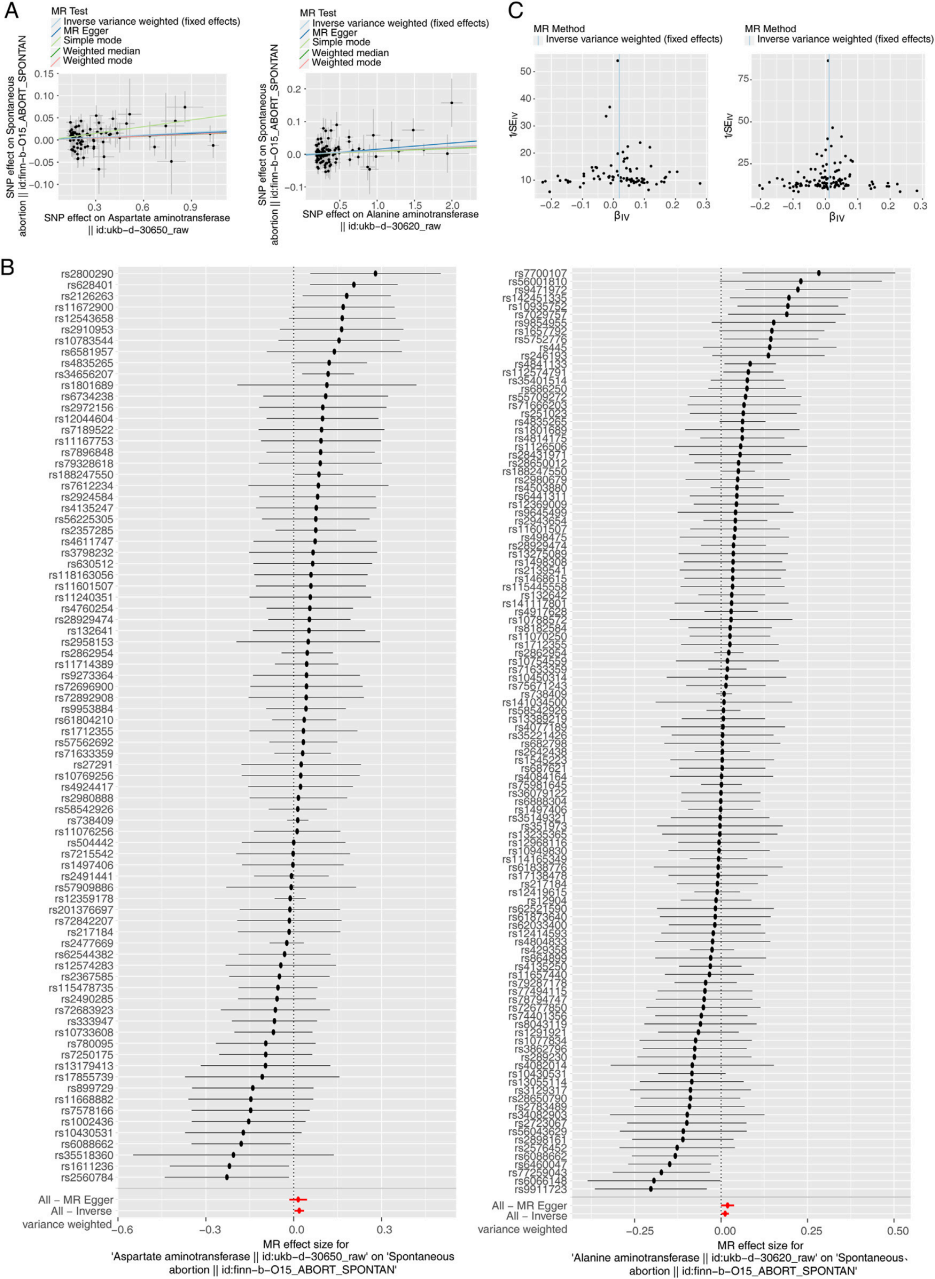
Causal effect of Aspartate Aminotransferase (AST) and Alanine Aminotransferase (ALT) on Spontaneous Abortion (SA) in forward Mendelian randomization (MR) analysis. **(A)** Scatter plot of genetic associations between exposure and outcome. Left: AST and SA. Right: ALT and SA. **(B)** Left: AST-SA funnel plot. Right: ALT-SA funnel plot. It is found that based on inverse variance weighted (IVW) method, almost left-right symmetry. **(C)** Left: The forest plot for the single nucleotide polymorphism (SNP) analysis of AST increasing on SA risk. Right: The forest plot for the SNP analysis of ALT increasing on SA risk. The *x*-axis shows the MR effect size for AST and ALT increasing on SA, while the *y*-axis illustrates the analysis for each of the SNPs. The dot and bar indicate the causal estimate and 95% confidence intervals (CI) of the association between AST and ALT increasing on SA risk.

After filtrating, there were 82 and 112 SNPs of AST and ALT successively acquired as IVs. All of these genetic variants had F statistics higher than 20, indicating a low likelihood of weak IVs (Supplementary Table 1).The forward MR results were presented in Supplementary Table 2. The causalities were detected between two aminotransferases and SA (AST: *P* = 0.013; ALT: *P* = 0.017), and all exposures were risk factors for SA (AST: odd ratio (OR) = 1.019; ALT: OR = 1.012) based on IVW method. The scatter plot also revealed that these exposures were risk factors on SA occurrence in accordance with the above MR results, less affected by confounding factors because of intercepts near zero (Figure 2A). Meanwhile, the diagnostic efficiency of each SNP of AST and ALT on outcome SA was estimated through MR Egger and IVW methods. The forest plots showed that the holistic points of AST and ALT were located in the right of baseline, supporting positive effects on the outcome (Figure 2B). The MR analysis of two exposures was in accord with Mendel’s second law random grouping (Figure 2C). In addition, reverse MR analysis Supplementary Table 3 showed that SA had no significant causal relationship with either of the two aminotransferases, in which AST and ALT as outcomes and SA as an exposure factor (*P* > 0.05).

### 3.2 Sensitivity analysis illustrated the reliability of the MR results

**FIGURE 3:**
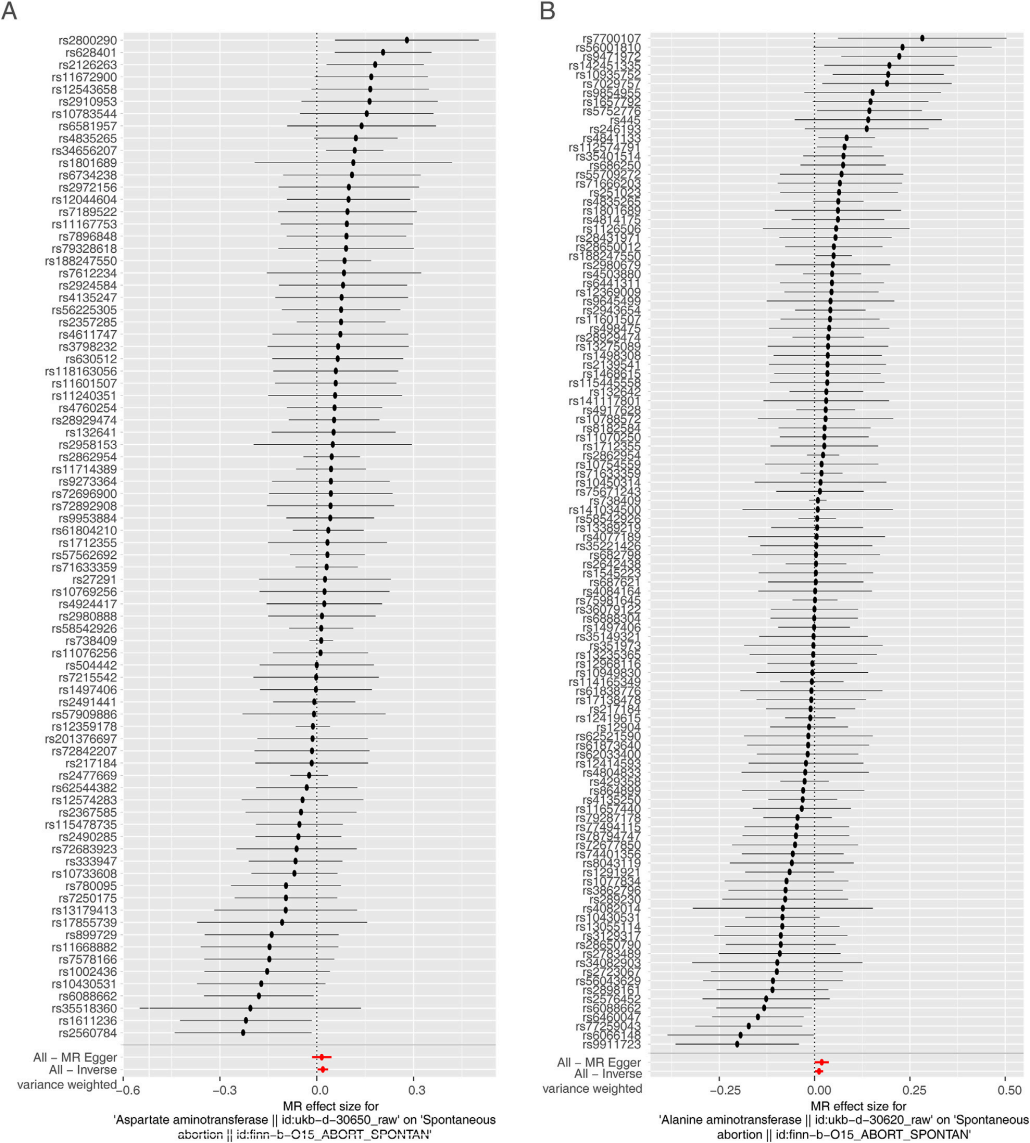
Sensitivity analysis. **(A)** Presentation of the leave-one-out sensitivity analysis for the effect of AST increasing SNPs on SA risk in the context of MR. **(B)** Presentation of the leave-one-out sensitivity analysis for the effect of ALT increasing SNPs on SA risk in the context of MR. The dot and bar indicate the estimate and 95% CI when a specific SNP is removed. OR, odds ratios; IV, instrumental variant; SE, standard error.

Furthermore, the reliability of the above MR results was demonstrated by the sensitivity analysis. There were no heterogeneity for AST and ALT via fixed effects IVW method (AST: Q_pval = 0.172, ALT: Q_pval = 0.086) (Table 1). The Pleiotropy test suggested there was no horizontal pleiotropy for two exposures (AST: *P* = 0.786; ALT: *P* = 0.370) with the help of the mr_pleiotropy_test function (Table 2). Finally, the influence of the remaining IVs on the outcome was evaluated after removing the IVs one by one. We found that there were no points of severe bias by LOO method (Figure 3). In conclusion, AST and ALT were risk factors on SA occurrence with the proven dependability.

**TABLE 1:** The heterogeneity test results of AST and ALT causal influence on SA.

id.exposure	id.outcome	Outcome	Exposure	Method	Q	Q_df	Q_pval
ukb-d-30650_raw	finn-b-O15_ABORT_SPONTAN	Spontaneous abortion || id:finn-b-O15_ABORT_SPONTAN	Aspartate aminotransferase || id:ukb-d-30650_raw	MR Egger	92.86228	80	0.154
ukb-d-30650_raw	finn-b-O15_ABORT_SPONTAN	Spontaneous abortion || id:finn-b-O15_ABORT_SPONTAN	Aspartate aminotransferase || id:ukb-d-30650_raw	Inverse variance weighted	92.94872	81	0.172
ukb-d-30620_raw	finn-b-O15_ABORT_SPONTAN	Spontaneous abortion || id:finn-b-O15_ABORT_SPONTAN	Alanine aminotransferase || id:ukb-d-30620_raw	MR Egger	130.8819	110	0.085
ukb-d-30620_raw	finn-b-O15_ABORT_SPONTAN	Spontaneous abortion || id:finn-b-O15_ABORT_SPONTAN	Alanine aminotransferase || id:ukb-d-30620_raw	Inverse variance weighted	131.8441	111	0.086

Q, Q-statistic; Q_df, degrees of freedom.

**TABLE 2:** The horizontal pleiotropy test results of AST and ALT causal influence on SA.

id.exposure	id.outcome	Outcome	Exposure	egger_intercept	se	pval	Judge
ukb-d-30650_raw	finn-b-O15_ABORT_SPONTAN	Spontaneous abortion || id:finn-b-O15_ABORT_SPONTAN	Aspartate aminotransferase || id:ukb-d-30650_raw	0.001172	0.004295	0.785652	NO
ukb-d-30620_raw	finn-b-O15_ABORT_SPONTAN	Spontaneous abortion || id:finn-b-O15_ABORT_SPONTAN	Alanine aminotransferase || id:ukb-d-30620_raw	−0.00326	0.003623	0.370465	NO

se, Standard Error; pval, P-value.

### 3.3 Mutually connected with AST and ALT on SA occurrence

We estimated mutually the effects of AST and ALT on SA using multivariable MR. A total of 126 SNPs were acquired as IVs after filtrating, including 69 for ALT and 57 for AST. In addition, the F statistics for both exposure factors were greater than 20 (Table 3). The multivariable MR results were presented in Supplementary Table 4. In MVMR analysis, the relationship between ALT, AST and SA was also evaluated, and only ALT (OR = 1.018, P = 0.035) had a strong potential causal relationship with SA. Besides, no horizontal pleiotropy was found in the IVW-MVMR sensitivity analysis (p = 0.061) (Table 4).

**TABLE 3:** The F-statistics of IVs in multivariate Mendelian randomization (MVMR).

	Exposure 1	Exposure 2
F-statistic	25.48706186	32.36263158

**TABLE 4:** The horizontal pleiotropy test results of AST and ALT on SA in Multivariate MR analysis.

Qstat	Qpval
110.5527388	0.060582766

## 4 Discussion

This study identified a causal relationship between AST and ALT levels and SA. These two transaminases are risk factors for the development of SA, and their functional complementarity mitigates this causal relationship. This suggests an interconnected causality between AST and ALT concerning the outcome, indicating that they tend to function more complementarily rather than independently. Furthermore, sensitivity analyses confirmed the reliability of the MR results. It has been shown that nearly 3% of pregnancies are complicated by liver disease, which may result in adverse outcomes for mother and child, but more studies are needed to understand the epidemiology of pregnancy-related liver disease and to assess the long-term prognosis of maternity (Hemani et al., 2018). Additionally, obesity-induced liver function abnormalities can affect embryonic development and have long-term adverse effects on the offspring (Jha et al., 2023). Elevated liver enzymes due to cytomegalovirus infection during pregnancy can lead to miscarriage and congenital malformations in newborns (Soares et al., 2018). Previous studies have also examined related topics, such as, studies have confirmed that significantly elevated serum transaminase levels in patients with recurrent miscarriage are an independent risk factor for early pregnancy failure (Zhang et al., 2023).

The pregnancy outcomes in women with hepatitis C are closely tied to liver enzyme levels (Llovet et al., 2019). Specifically, ALT (alanine aminotransferase) serves as a critical prognostic indicator in pregnant women with COVID-19 (Carrión-Nessi et al., 2023), and serum ALT levels are a key factor in determining pregnancy outcomes in women with hepatitis B virus infection during pregnancy (Nguyen et al., 2009; Alves and Rapp, 2023). The underlying mechanisms appear to involve two primary aspects. First, the AST (aspartate aminotransferase)/GOT (glutamate oxaloacetate transaminase) enzyme facilitates the transfer of amino groups from aspartate to alpha-ketoglutarate, resulting in the formation of glutamate and oxaloacetate. Substantial tissue destruction leads to the release of AST/GOT and ALT/GPT into the serum. Secondly, elevated serum levels of AST/GOT in pregnant women indicate that both AST and ALT play roles in oxidative stress. It has been determined that infection with the hepatitis E virus (HEV) initiates an immune and inflammatory response in the body, resulting in damage to hepatocytes and elevated levels of AST and ALT. This inflammatory response may also compromise placental blood perfusion and alter the environment in which the fetus grows and develops, thereby increasing the risk of miscarriage (Qian et al., 2023).

This study’s main finding is the identification of a causal link between ALT and AST levels and spontaneous abortion (SA). Fluctuations in ALT and AST levels appear to elevate the risk of SA. However, after conducting additional statistical analyses through univariate and multivariate Mendelian randomization (UVMR and MVMR) and assessing the multivariate level, the inverse variance weighted (IVW) algorithm confirmed a positive causal relationship between AST/ALT levels and spontaneous abortion (SA). Conversely, the reverse MR analysis revealed no significant causality between SA and either aminotransferase. This result is not contradictory but rather illustrates the utility and limitations of MR analysis in determining the direction of causal inference. ALT remained a significant risk factor for SA, whereas AST’s significance diminished. This outcome may be due to a covariance or interaction between ALT and AST, obscuring AST’s independent effect. Additionally, other unaccounted-for confounders or potential common causes might interfere with the effects of ALT and AST, rendering the impact of AST insignificant.

The findings underscore the importance of monitoring ALT levels in assessing the risk of SA, particularly in individuals with related risk factors. The study also sheds light on the interconnected causal relationship between AST and ALT, suggesting a collaborative role in the pathogenesis of SA and paving the way for future mechanistic research. These insights advocate for the routine monitoring of ALT levels, especially in those at risk, to better understand and mitigate SA risk.

However, it is important to acknowledge that this study has certain potential limitations and pitfalls. Firstly, whether the population studied is representative of the population as a whole, and if the study sample is not representative, then the external generalizability of the findings may be limited. Hence, future studies should consider expanding sample size and including more diverse populations to ensure that findings are more generalizable. Another important consideration is the potential influence of confounding factors. By analyzing multiple exposures simultaneously, multivariate Mendelian randomization (MVMR) can partially control for confounders linked to various exposures. Although our study has diligently attempted to account for the effects of multiple exposure factors on outcomes through MVMR methods, it remains true that several potential confounders might not have been directly measured. These unmeasured confounders could include, but are not limited to, non-genetic environmental factors such as lifestyle, dietary habits, socioeconomic status, measurement errors, and possible gene-environment interactions. Furthermore, in our multivariate analyses, the influence of the two transaminases on the outcome appeared diminished. This attenuation may result from the multivariate context, where interactions and covariance among variables may diminish the apparent effect of any single variable. To more comprehensively assess the impact of potential confounders, we plan to further investigate and control for these factors in future studies, employing advanced techniques that incorporate broader genetic and environmental data, as well as more refined statistical models. The databases used in this study predominantly encompass genetic information from European men and women. Given that genetic variations across different races and ethnicities, as well as environmental factors such as dietary habits, lifestyle, and exposure to pollutants, can significantly affect liver function indicators and overall health status, this may restrict our ability to generalize the findings concerning the relationship between AST/ALT levels and spontaneous abortion (SA). To overcome this limitation, future studies will aim to incorporate more diverse datasets from various races, ethnicities, and geographic regions to more thoroughly evaluate the combined effects of genetic variants and environmental factors on AST/ALT levels and SA.

Additionally, issues such as pleiotropy and linkage disequilibrium could potentially complicate Mendelian Randomization (MR) analyses, affecting the accuracy of causal inferences. To address these concerns, comprehensive sensitivity analyses were conducted, and their results supported the reliability of our primary conclusions. We anticipate further refining MR analysis techniques in subsequent studies to enhance both the accuracy and reliability of causal determinations.

While this study offers insights into biomarker discovery and potential therapeutic targets, the absence of direct clinical validation and longitudinal data means we cannot definitively ascertain the clinical significance of AST/ALT or other potential biomarkers in managing spontaneous abortion. This represents a significant research gap and will be a primary focus moving forward.

Future efforts could include more extensive clinical validation and longitudinal studies. Present basic research remains crucial for understanding the pathophysiological mechanisms behind spontaneous abortion, discovering new biomarkers, and identifying therapeutic targets. This work lays a vital theoretical foundation and provides a direction for upcoming clinical research and interventions. Finally, there is the long-term follow-up. This study may have only covered data over a period of time, whereas SA may be associated with long-term lifestyle and health factors. Therefore, the need for long-term follow-up and research also needs to be emphasized. Long-term follow-up studies are essential to verify the durability of research findings over time and to gain a deeper understanding of the enduring effects of relevant variables on health outcomes.

Research has indicated that levels of AST/ALT and HSI are linked with the increased risk of hypertensive disorders of pregnancy (HDP) (Liu et al., 2024). Additionally, the serum levels of biochemical markers such as TBA, DBIL, and ALT in pregnant women diagnosed with intrahepatic cholestasis of pregnancy (ICP) subtypes at the onset of pregnancy are subject to continual variation, mirroring changes in the subtypes and severity of ICP. These fluctuations can result in numerous adverse pregnancy outcomes (Feng et al., 2024).

Moreover, either isolated or widespread elevations in liver enzyme levels, particularly notable increases in gamma-glutamyltransferase (GGT), alkaline phosphatase (ALP), and aspartate aminotransferase (AST) during early pregnancy, have been identified as three crucial independent predictors for the onset of gestational diabetes mellitus (GDM) (Cui et al., 2024). Variations in these liver enzymes are associated not only with GDM but can also significantly influence the long-term risk of chronic conditions such as metabolic syndrome in pregnant women. We aim to investigate the specific mechanisms behind elevated liver enzymes during pregnancy, including genetic and environmental factors and their interactions. We will also analyze how changes in liver enzymes affect the mother’s future risk of metabolic syndrome, diabetes mellitus, and other chronic illnesses. Additionally, we plan to evaluate whether these changes heighten the risk of metabolic or cardiovascular diseases in the offspring, thereby elucidating the complexity and diversity of liver enzyme alterations during pregnancy.

In response to the current findings, it is known that there may be an interaction between AST and ALT in the pathogenesis of SA, and future studies could further explore the mechanism of this interaction to understand their mutual effects in inflammation, fat metabolism, and liver injury, which could theoretically provide a more in-depth theoretical basis for this finding.

## 5 Conclusion

In conclusion, the present study provides important preliminary evidence for a causal relationship between transaminases (AST and ALT) and SA and highlights their interrelationship. However, further studies are needed to address potential limitations, and in future studies, potential constraints need to be addressed, potential roles of genetic and environmental factors between aminotransferase levels and SA need to be explored, and biological mechanisms linking aminotransferase levels to SA need to be studied in depth. In the meantime, continued attention to progress in the study of SA and these two aminotransferases will have significant clinical and SA reduction implications.

## Data Availability

The raw data supporting the conclusions of this article will be made available by the authors, without undue reservation.

## Abbreviations

AST, Aspartate Aminotransferase; ALT, Alanine Aminotransferase; SA, Spontaneous Abortion; MR, Mendelian Randomization; GWAS, Genome-wide association study; IVW, Inverse Variance Weighted; LOO, Leave-One-Out; MVMR, multivariable MR; OR, odd ratio; IEU, Integrative Epidemiology Unit; SNPs, single nucleotide polymorphisms; IVs, instrumental variables; CI, confidence interval; SE, standard error.
